# Pulsed FCAW of Martensitic Stainless Clads onto Mild Steel: Microstructure, Hardness, and Residual Stresses

**DOI:** 10.3390/ma15082715

**Published:** 2022-04-07

**Authors:** Joao Sartori Moreno, Fabio Faria Conde, Celso Alves Correa, Luiz Henrique Barbosa, Erenilton Pereira da Silva, Julian Avila, Ricardo Henrique Buzolin, Haroldo Cavalcanti Pinto

**Affiliations:** 1 Mechanical Department, Federal Technological University of Paraná, Cornelio Procópio 86300-000, Brazil; jrs.more12@gmail.com (J.S.M.); celsoalvescorrea2014@gmail.com (C.A.C.); 2 Materials Engineering Department, São Carlos School of Engineering, University of São Paulo, São Carlos 13563-120, Brazil; fabiofariaconde@gmail.com (F.F.C.); haroldo@sc.usp.br (H.C.P.); 3 Institute of Engineering, Science and Technology, Federal University of Vales do Jequitinhonha e Mucuri, Janaúba 39440-000, Brazil; luiz.barbosa@ufvjm.edu.br (L.H.B.); erenilton.silva@ufvjm.edu.br (E.P.d.S.); 4 Campus of São Joao da Boa Vista, São Paulo State University—UNESP, São Joao da Boa Vista 13870-000, Brazil; julian.avila@unesp.br; 5 Christian Doppler Laboratory for Design of High-Performance Alloys by Thermomechanical Processing, Kopernikusgasse 24, 8010 Graz, Austria; 6 Institute of Materials Science, Joining and Forming, Graz University of Technology, Kopernikusgasse 24/I, 8010 Graz, Austria

**Keywords:** cladding, FCAW, residual stresses, mechanical properties, martensitic stainless steel

## Abstract

The low carbon martensitic stainless AWS 410NiMo steel has in its chemical composition 13% chromium, 4% nickel, and 0.4% molybdenum (wt.%) and is used in turbine recovery, rotors, and high-pressure steam pump housings due to its resistance to impact at low temperatures, as well as to corrosion and cavitation. Those applications of the AWS 410NiMo steel frequently demand repair, which is performed by welding or cladding. Arc welding is a well-established technique for joining materials and presents several parameters that influence the mechanical performance of the weld bead. Although numerous welding processes exist, optimizing welding parameters for specific applications and materials is always challenging. The present work deals with a systematic study to verify the correlation between the pulsed fluxed core arc welding (FCAW) parameters, namely pulse current and frequency, welding speed, and contact tip work distance (CTWD), and the bead morphology, microstructure formation, residual stress, and hardness of the martensitic clad. The substrate used was the AISI 1020 steel, and the AWS 410NiMo steel was the filler metal for clad deposition. From the initial nine (9) samples, three (3) were selected for in-depth characterization. Lower heat input resulted in lower dilution, more elevated hardness, and lower compressive residual stresses. Therefore, the results highlight the need for selecting the proper heat input, even when using a pulsed FCAW procedure, to achieve the desired performance of the clad. In the present case, a higher heat input appears to be more advantageous owing to the lower convexity index, smooth hardness transition between fusion and heat-affected zones in addition to more elevated compressive stresses.

## 1. Introduction

The main component of a hydroelectric plant, the hydraulic turbine, converts the waterfall’s kinetic energy into mechanical energy, which is converted by a generator into electrical energy. These components are subjected to various mechanical and chemical stresses and are mainly worn by cavitation [1]. The hydraulic units that make up the hydropower plants are equipped with large turbine runners made of low carbon steel, 9 m in diameter, and mass up to 300 tons. These runners are typically engineered for service lifetimes of 70 years.

The turbine runners were initially manufactured from C-Mn steel castings for structural applications [2], classified according to ASTM A27 [3]. According to ASTM A743 [4], the replacement of C-Mn steels by soft martensitic stainless steels, cast with 13% Cr, 4% Ni, and 0.4% Mo and classified as CA6NM, are associated with better resistance to corrosion and to damage by cavitation, combined with good weldability, when compared to other martensitic stainless steel grades, because the reduced carbon content improves toughness and weldability [5]. Cast C-Mn steels exhibit a 250 MPa yield limit. In comparison, cast CA6NM stainless steels have a minimum yield limit of 550 MPa, allowing the design and construction of hydraulic turbine components with better operating efficiency, less thickness, and lower operating conditions [6]. Thus, the CA6NM steel grade became relevant mainly for hydropower and turbine parts, welding, or clads.

These turbine runners are subjected to critical cyclic loads generated from the transient operation during service, slurry erosion resultant from the repeated impact of solid particles carried by liquids, and complex hydraulic phenomena, such as high-frequency pressure fluctuations [7,8,9]. For these reasons, cladding is applied in the manufacturing process since it increases the resistance to wear and cavitation [10,11]. Recently, several hybrid organic-inorganic materials have been proposed to enhance the corrosion resistance of metallic substrates using thin films manufactured by solution-based and electrolytic processing or chemical/physical vapor deposition, in addition to atomic or molecular deposition technologies [12,13]. However, when wear processes occur concomitantly with corrosion, material degradation is accelerated, and coating thickness plays a major role. Hence, claddings produced by welding and additive manufacturing technologies become competitive.

Powder bed fusion (PBF) processes [14,15] using a laser or electron beam are the first alternative to melt powder together and build up thicker wear and corrosion-resistant coatings. As the raw material is a powder, the manufactured workpieces have better finishing, and thus, more complex geometries can be produced. However, PBF takes a long time for manufacturing large parts. Direct energy deposition (DED) [16,17] is another form of additive manufacturing that uses a powder or metal wire along with the energy source to add or melt a material onto an existing part or to create a new part. DED is faster than other methods for cladding and is extremely accurate. Some disadvantages are the use of large volumes of inert gas in laser-based DED, whereas the electron-based system requires vacuum. In DED, the deposition efficiency is typically high when the metal wire is used compared to powder deposition since only a small amount of metal powder must be melted [15]. The disadvantages, as mentioned earlier, of powder/wire-based technologies using a laser or electron beam can be overcome by wire arc cladding that offers additional advantages, such as minimal wastage of material, high flexibility of equipment, low running costs, short-time production cycles, and excellent forming quality.

With the development of new electronic power supplies for arc welding, it is currently possible to use pulsed arc welding, which provides better control of the electric arc with less energy input, thus resulting in few distortions of the welded parts. Although the pulsed technique presents a high number of parameters, if adjusted correctly, it provides better weld quality than conventional arc welding [18].

The fluxed core arc welding process (FCAW) is a fusion welding process that employs tubular wire. The heat necessary for joining is provided by an electric arc established between the part and a continuously fed wire. The tubular wire has a flux composed of inorganic and metallic materials that exhibit several functions, among which the improvement of the characteristics of the electric arc, the transfer of the weld metal, the protection of the fusion bath, and in some cases, the addition of alloy elements, in addition to acting as slag former. The chemical composition of the flux also influences the mechanical properties of the deposited metal [5]. The application welding processes with tubular wires have grown in recent years due to high deposition rates and new consumables, which led companies to migrate to the tubular process compared to the SMAW and GMAW processes. The segments such as naval and offshore, heavy construction, welding of structural profiles, and industrial pipes for repair or maintenance are among those that most use welding with tubular wires [19]. However, FCAW presents some challenges as another welding process: storage of consumables and hydrogen embrittlement [20,21].

Although the welding process may be employed for cladding and repairing components back to their integrity, the weld bead geometry and properties play a vital role in determining the final mechanical properties of the entire workpiece. Therefore, the bead geometry can be used as a quality measure, i.e., bead width and height, depth of penetration, and convexity index (ratio between width and height). These features are dependent on welding parameters, such as wire feed rate, welding current, welding speed, plate thickness, base current (Ib), peak current (Ip), base time (tb), and peak time (tp). Therefore, it is essential to set up proper welding parameters to produce adequate process stability and suitable weld bead geometry [5,22].

Recent works [23,24,25] have shown the cladding efficiency achieved with martensitic stainless steel in hydraulic turbine generators. These hard materials present high toughness, excellent resistance to cavitation and erosion, good hardenability, and weldability. However, the microstructural changes in the base metal when receiving the clad are fundamental to obtaining an adequate adherence to the substrate and warrant adequate wear and corrosion resistance at the surface of the workpiece. Considerable hardness fluctuations across the partially diluted zones (PDZ) along with residual tensile stresses favor crack initiation and brittle failures during service and a consequent reduction in the cladded component life.

The present work deals for the first time with the interplay of the pulsed FCAW process variables (average current, pulse frequency, welding speed, and contact tip work distance—CTWD) with fundamental microstructural features, such as phase formation and distribution, weld bead morphology, residual stresses, and the resulting mechanical properties of the clad. AISI 1020 steel was applied as substrate, and the low carbon martensitic stainless steel 410NiMo was used for cladding. The evolution of the base metal microstructure, heat-affected zones (HAZ), diluted zones, and bead on plate clads with different deposition conditions was systematically discussed using optical and electron microscopy, electron backscatter diffraction (EBSD), X-ray stress analyses, and micro-hardness profiles. The relevance of adjusting the welding heat input by understanding the microstructure formation and the resulting mechanical properties is demonstrated.

## 2. Materials and Methods

Plates of AISI 1020 steel (GERDAU Aços Especiais, Londrina/PR, Brazil) with dimensions of 185 × 63.5 × 12.7 mm were used as the substrate for welding. This steel is widely used in gears, camshafts, and bolts due to its ease of machining and ductility. A corrosion-resistant 410NiMo (~13% Cr, 0.5% Mo) (ESAB, Contagem/MG, Brazil) was selected as cladding material. The filler metal was the AWS EC410NiMo tubular wire with 1.2 mm diameter (ESAB, Contagem/MG, Brazil). Table 1 lists the chemical composition of these materials, as reported in the specification data sheets provided by their manufacturers.

**Figure 1:**
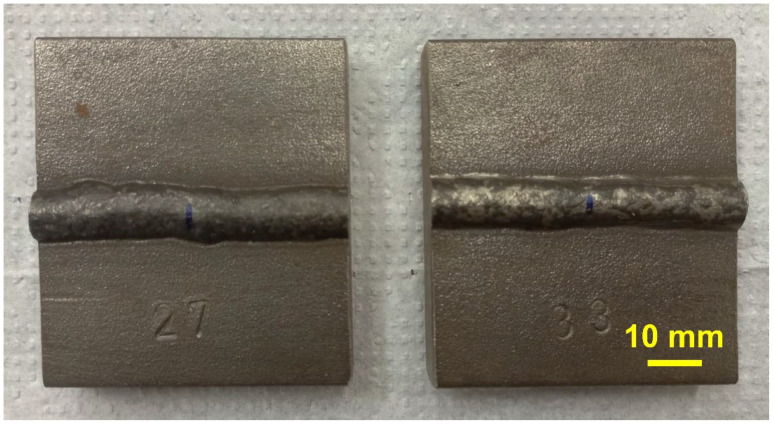
Macroscopic image of two bead-on-plate clads produced by pulsed fluxed core arc welding process (FCAW).

**Figure 2:**
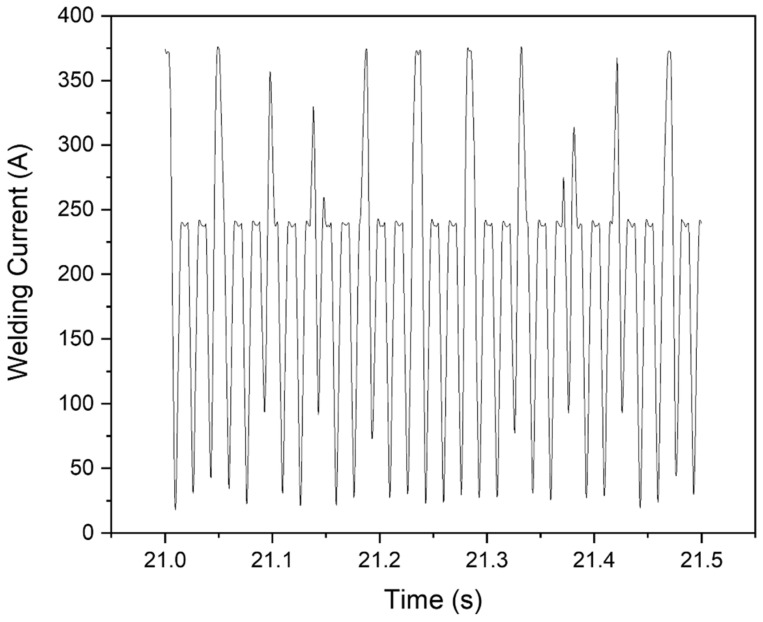
Example of oscillogram displaying pulsed welding current registered over time for cladding 410NiMo stainless steel onto AISI 1020 steel using FCAW at a mean current of 200 A.

An electronic multiprocess inversal 450A IMC (IMC Soldagem, Florianopolis/SC, Brazil) welding power supply was used for depositing beads using pulsed current. All specimens used were free of grease, oil, and contaminants. They were heated in a furnace at 200 °C, taken to the welding bench, and welded when the temperature was 150 °C. That procedure was applied to all the samples. The pulsed FCAW process was used, and the following welding conditions were kept constant: a weld bead with a mean peak current of 305 ± 60 A, peak time of 10 ms, wire feed rate of 8.5 m·min^−1^, a direct current positive electrode, Ar + 2 vol.% O_2_ shielding gas with a flow rate of 18 L·min^−1^. The stable arc length was 2.0 mm. The welding position consisted of 1G/PA, and the torch was fixed at 90 degrees. Figure 1 displays macroscopic images of two bead-on-plate clads produced in this work. Figure 2 illustrates an oscillogram showing the variation of the pulsed welding current with time for cladding 410NiMo stainless steel onto the AISI 1020 substrates at a mean current of 200 A.

After conducting preliminary tests using as variables the average pulse current, the pulse frequency, the welding speed, and the nozzle contact distance piece, three levels were chosen for all process parameters. Due to the numerous variables, the Taguchi method was a practical approach to obtain the correlation among parameters, reducing the number of experiments [26]. Therefore, the experimental design adopted was the Taguchi ‘L9′ method [26], and Table 2 shows the variables and levels; nine (9) samples were investigated. When performing the tests, the test sequence was randomized to minimize the uncertainties caused by the equipment and operators. The Minitab 17 software was used to conduct the statistical analysis.

The heat input Q in J·mm^−1^ was estimated with Equation (1) from the ISO/TR 17671-1 [27]. U corresponds to the arc voltage in V, I the arc welding current in A, and v is the welding speed in mm·s^−1^. The K factor was equal to 0.8 because the same standard is recommended to FCAW. The mean current value (I_m_) and root mean square (I_rms_) value of current can be measured during welding, and both are related to the weld bead formation [28]. Furthermore, there is a relationship between I_m_, welding penetration, I_rms_, and the weld width [29].

(1) $$Q=k\;\frac{U\times I_{\mathrm{rms}}}{v}$$

### 2.1. Macrostructure and Microstructure

The samples were cut near half of the weld bead’s length. They were metallographically prepared using a standard procedure of mechanical grinding and polishing steps, according to the ASTM E3 standard recommendations [30]. The etching was performed with a 2 vol.% HNO_3_ solution (Nital 2%) for 10 s. The weld beads were analyzed in terms of geometry, surface visual appearance, and microstructural observation. The regions analyzed in the deposited clad were the base metal (BM), heat-affected zone (HAZ), fusion zone (FZ).

**Figure 3:**
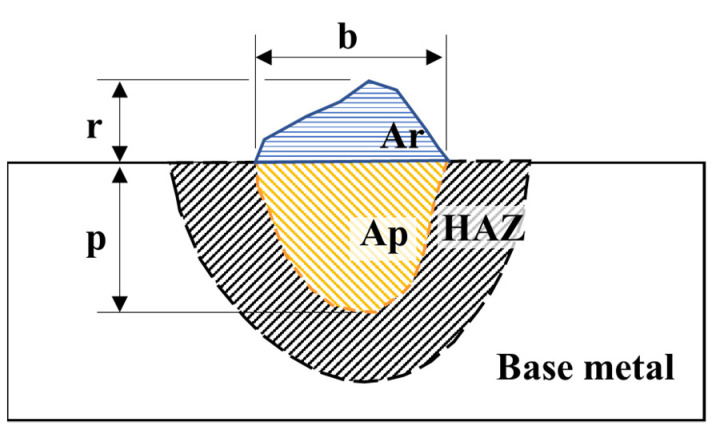
Schematic cross-section of the weld bead. Where: reinforcement height (r); bead width (b); penetration depth (p); reinforcement area (Ar); penetration area (Ap), and heat-affected zone (HAZ).

The measurements of the reinforcement height (r), bead width (b), and penetration depth (p) of the weld beads, as shown in Figure 3, were conducted on macrographs taken with an optical microscope. The convexity index (CI) and dilution (D) were calculated according to Equations (2) and (3). In addition, crystallography features were studied using EBSD. Finally, residual stresses were evaluated using X-ray diffraction (XRD).

CI = (r/b) × 100(2)

D = (Ap/(Ar + Ap)) × 100(3)

The electron microscopy work was conducted with the SEM/EBSD FEI^®^ Quanta 650 FEG (Hillsboro, Oregon, United States) and a Tescan^®^ Mira3 (Brno, Czech Republic). Backscattered electron (BSE) micrographs and energy dispersive spectroscopy (EDXS) analysis were carried out using an acceleration voltage of 20 kV, with a working distance of 15 mm, and a spot size of 30 nm. In addition, the EBSD measurements were carried out using an acceleration voltage of 20 keV and a step size between 0.2 and 2.0 µm. The EBSD data were analyzed using the HKL Channel 5 system from Oxford (Abingdon, United Kingdom). Large maps showing the transition between BM, HAZ, and FZ were measured with a step size of 0.75 and 2 µm. In addition, localized maps with a step size of 0.2 µm were used to indicate the type of microstructure, grain morphologies, and boundaries.

### 2.2. Hardness Measurements

The test specimens were removed from the central region of the weld bead. Samples were also conventionally prepared metallographically and etched with a 4% Nital etching for 1 min to reveal the fused zone, the heat-affected zone, and the base metal.

**Figure 4:**
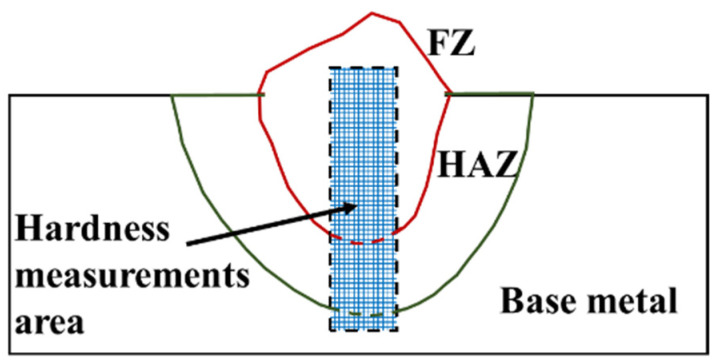
Schematic representation of hardness impressions of the weld bead. Where: heat-affected zone (HAZ) and fused zone (FZ).

Vickers hardness linear profiles were made using the Leco LM100-AT (St. Joseph, Michigan, USA) hardness system with a 5 N load and 200 µm indentation distances. The hardness profiles were measured through the base metal (BM), heat-affected zone (HAZ), and fusion zone (FZ), as shown in Figure 4 and can be correlated to the microstructural evolution during welding in the clad.

### 2.3. Residual Stresses

**Figure 5:**
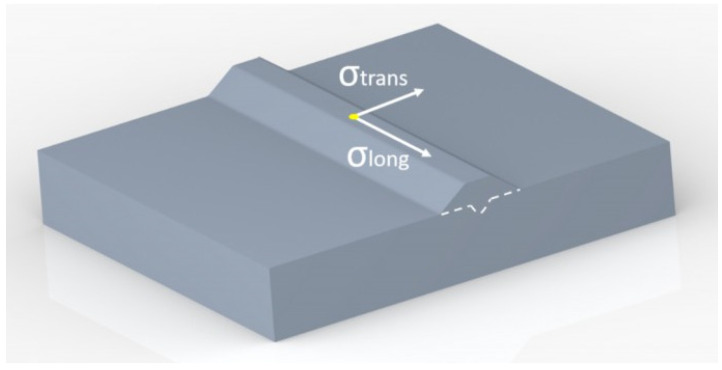
Residual stress components determined by X-ray diffraction and the location of measurement. σ_trans_ and σ_long_ correspond to the transversal and longitudinal stresses, respectively.

Samples of the 410NiMo stainless steel clad with 58 mm in length and 4.4 mm in width were prepared for residual stress analysis using X-ray diffraction. The longitudinal and transversal stress components were determined using a PANALYTICAL MRD-XL (Malvern, Worcestershire, United Kingdom; Almelo, Netherlands) diffractometer equipped with Co-K_α_ radiation (1.78897 Å wavelength) and using a beam size of 2 mm × 2 mm. Only one point of interest was measured on each sample at the midpoint along the weld bead top surface. Figure 5 shows schematically the location and stress components analyzed by X-ray diffraction. The lattice strains were calculated for the (211) diffraction line of martensite and converted to stresses by following the sin^2^ψ method covering a sin^2^ψ-range from 0 to 0.8 and using a step size of 0.16 in sin^2^ψ. The diffraction elastic constants (DEC) were calculated based on the Eshelby–Kroener approach [31].

## 3. Results

### 3.1. Macro and Microstructural Analysis

Table 3 shows the results of weld beads, the height of the weld bead (r), the width of the weld (b), penetration (p), showing two measurements, and an average of each bead (rAV, bAV, and pAV). The convexity index (CI) was calculated with the average of the width and reinforcement, according to Equation (1). Table 3 shows that the lowest convexity index was 30.55%, the maximum 52.36%, and the average of all samples was 38.73%.

Wide weld beads in clads are recommended to reduce the number of passes, thus increasing the process’s productivity. However, the recommended values for CI should be around 30% [5,22]. The clad’s mechanical strength is compromised when the convexity index is greater than 30%. As a result, early fracture failure may occur when subjected to mechanical solicitation on the weld bead’s surface. Conversely, productivity is reduced when this index is much smaller than 30%. Consequently, much more passes are required to reach the desired layer height, increasing the manufacturing process time [5].

For each level of average welding current (170, 200, and 230 A), the test closer to 30% in the convexity index was chosen to perform the microstructural analyses and residual stress. For the average current of 170 A, sample 2 was selected with a convexity index of 36.80%. Sample 6 was selected for the current of 200 A with a convexity index of 33.95%. Finally, sample 9 was selected for the current of 230 A with a convexity index of 30.55%. The heat input increased from samples 2 to 9. Table 4 shows the schematic macro view of the cross-section of the selected samples and the welding process parameters, as shown in Table 2 and Table 3.

**Figure 6:**
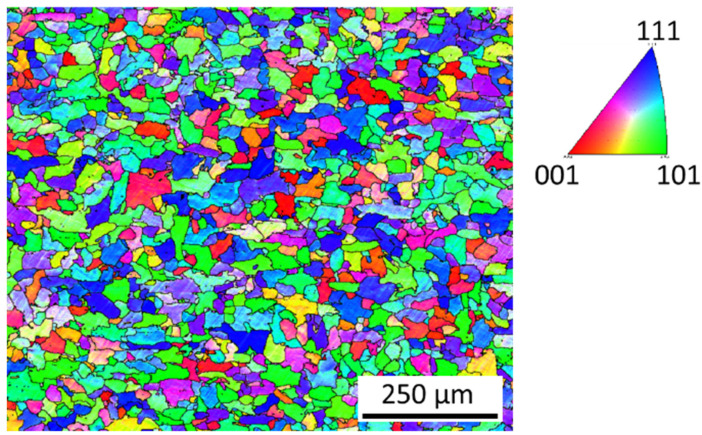
Inverse pole figure (IPF) map of the AISI 1020 base metal.

Quasi-polygonal ferrite with grain size around 30 µm forms the microstructure of the AISI 1020 steel substrate. The microstructure is shown in the inverse pole figure (IPF) map in Figure 6, with an average grain size of 36 ± 10 µm.

**Figure 7:**
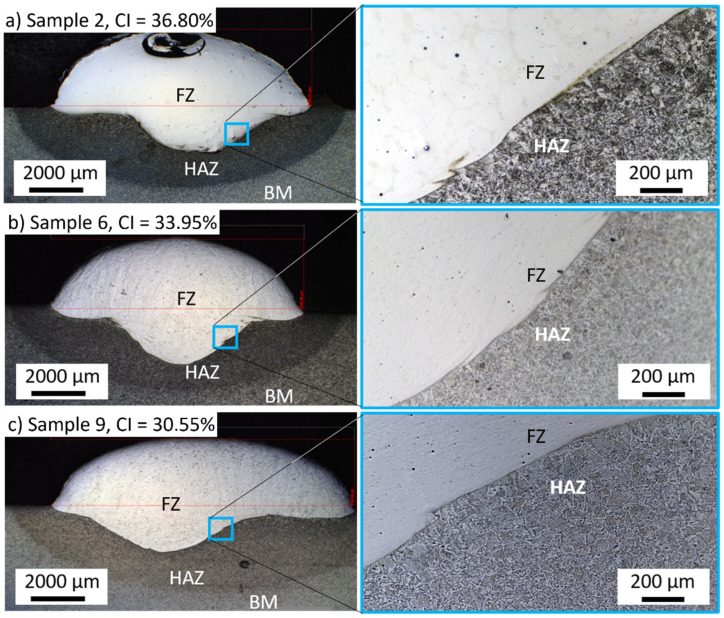
Cross-section of the weld beads for (**a**) sample 2, (**b**) sample 6, and (**c**) sample 9. The fusion zone, heat-affected zone, and base metal are indicated by FZ, HAZ, and BM, respectively.

Figure 7 shows optical micrographs of the cross-section of samples 2, 6, and 9. The CI decreased from samples 2 to 9. The combinations of welding parameters caused variation of penetration depth, i.e., the distance that the fusion extended into the base metal from the substrate surface after welding. Sample 6 had the deepest penetration, 2.28 mm, while sample 2 had a penetration of 1.79 mm. A penetration of 2.19 mm was reached for sample 9. The higher the heat input produced, the wider the deposition. The highest heat input produced the widest deposition in sample 9, and the thinnest was sample 2, with the lowest heat input.

In Figure 7, the heat-affected zone (HAZ) near the fused zone (FZ) shows a lath-type morphology, similar to the martensitic structure. Sample 2 showed a more pronounced lath-type morphology at the HAZ than conditions 6 and 9. The martensite laths were coarse near the fusion line and became finer towards the base metal (BM).

**Figure 8:**
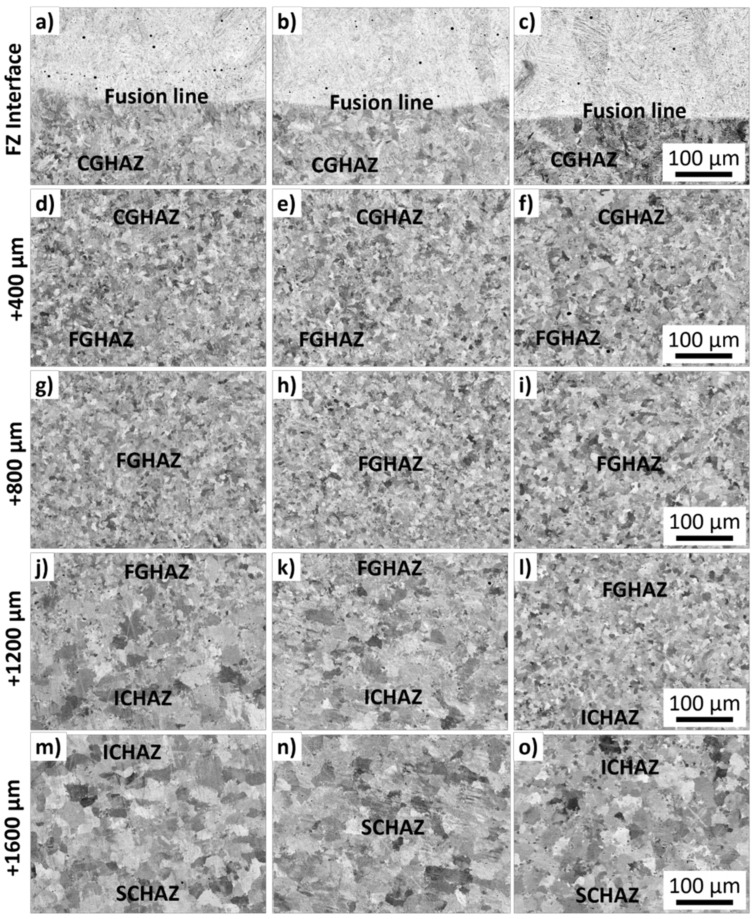
Scanning electron microscopy (SEM) backscattered electrons (BSE) micrographs with the microstructure of studied welded steels. From left to right, columns show samples 2, 6, and 9. At each line, the regions are further ~400 µm apart. The fusion zone (FZ) (**a**–**c**), the coarse-grained heat-affected zone (CGHAZ) (**d**–**f**), the fine-grained heat-affect zone (FGHAZ) (**g**–**i**), the inter-critical heat-affect zone (ICHAZ) (**j**–**l**), and the subcritical heat-affect zone (SCHAZ) (**m**–**o**) are indicated. A total distance of 1600 µm is shown.

Figure 8 shows the BSE micrographs of different regions of the produced clads for samples 2, 6, and 9. The fusion lines are indicated in Figure 8a–c. Large inclusions or secondary phases were not visible in any investigated regions. The microstructure of all samples consisted of similar metallurgical areas typical of fusion cladding materials. A dendritic-like form the fusion zone (FZ), while a lath-type microstructure organized in colonies formed the coarse-grained heat-affected zone (CGHAZ), the fine-grained heat-affect zone (FGHAZ), the inter-critical heat-affect zone (ICHAZ), and the subcritical heat-affect zone (SCHAZ).

**Figure 9:**
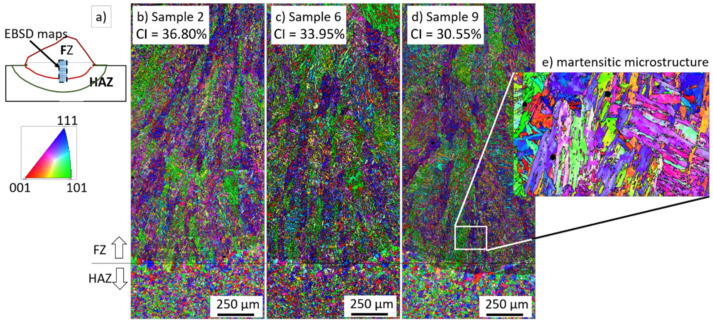
EBSD results of the FZ and HAZ, mainly the upper part, the CGHAZ: (**a**) schematic view where the macros view were taken, (**b**) IPF map of samples 2, (**c**) IPF map of sample 6, (**d**) IPF map of sample 9, (**e**) detailed view from sample 9, showing the formation of martensite blocks and laths inside the microstructure.

Figure 9 shows the IPF maps of the FZ and HAZ for samples 2, 6, and 9, as shown schematically in Figure 9a. The CGHAZ mainly represents the HAZ. Despite the differences in CI and heat input, large martensitic blocks with similar morphology and crystallographic orientation in the FZ were formed for the three investigated samples. Figure 9e shows the martensitic microstructure of a lath-type morphology inside the martensite blocks.

Different metallurgical zones were formed in the weld bead due to the effect of the heat input on the recrystallization of the microstructure and phase transformation. A well-defined FZ, a CGHAZ, an FGHAZ, an ICHAZ, and a SCHAZ were observed for all conditions. No considerable morphological differences were observed for the ferrite and martensite phases for the investigated samples 2, 6, and 9. The FZ consisted of a needle-like mixed with a lath-type morphology, Figure 9e. The non-indexed regions (dark regions) in Figure 9e correspond to inclusions from the wire fluxes formed inside the FZ.

**Figure 10:**
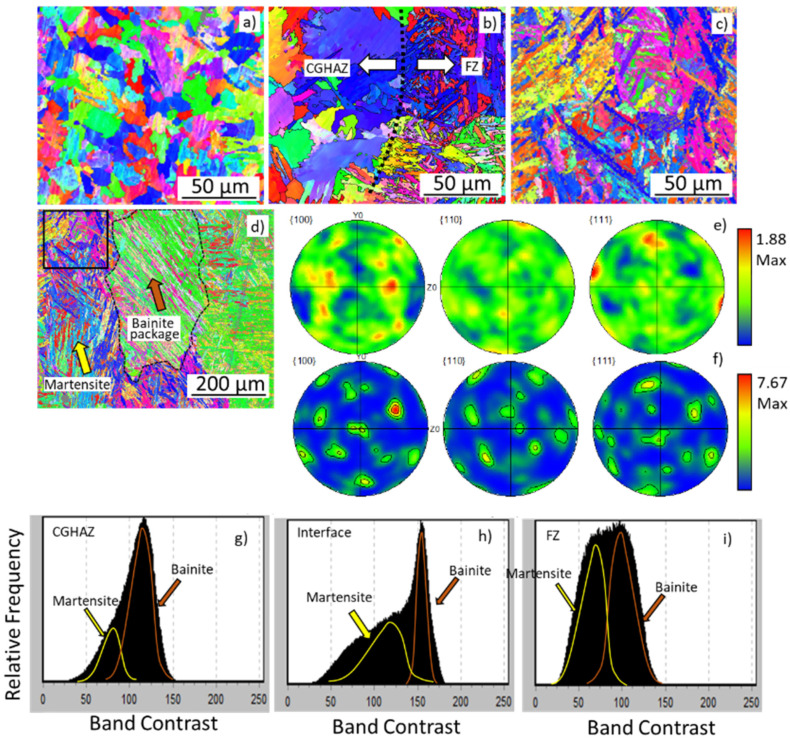
EBSD results of the formed metallurgical zones in the weld bead: (**a**) inverse pole figure (IPF) of the CGHAZ near fusion line; (**b**) CGHAZ and FZ interface; (**c**) high magnification of black square in (**d**); (**d**) FZ in lower magnification; (**e**) pole figure (PF) of the CGHAZ; (**f**) PF of the FZ. Band contrast (BC) with the convolution of martensite and bainite peaks for the (**g**) CGHAZ, (**h**) FZ interface, and (**i**) FZ.

Figure 10 shows in detail the microstructure of the formed metallurgical zones. Figure 10a shows the formation of the CGHAZ with an equiaxed and polygonal microstructure, typical of bainite and ferrite phases. The mean grain size in the CGHAZ was ~40 µm. Figure 10b,c shows the microstructure of the FZ with a strong appearance of the needle and lath-type structures, characteristic of martensite formation [32]. Figure 10d shows a low magnification view of FZ, where a package morphology of clusters was also formed, typical of bainite formation. The BC from EBSD data allows differentiating and estimating the fractions of martensite, bainite, and polygonal ferrite, which are found in this order with an increase in the BC value [33,34,35]. Band contrast (BC) of the FZ in Figure 10i confirms the convolution of two peaks indicating martensite and bainite formation. The peak had nearly the same area, meaning a similar martensite volume and bainite. Differently, the CGHAZ (Figure 10g) had a BC with a much smaller area for the martensitic peak compared to the bainitic one. The weld line interface (Figure 10h) had an amount of martensite formation intermediate between the CGHAZ and the FZ. The pole figures (PF) (Figure 10e) revealed the random crystallographic orientation at the CGHAZ, while the FZ (Figure 10f) had a defined texture. The PF of the FZ (Figure 10f) showed 001 mainly aligned at 60° from the normal direction and with the intensity of 7.67 multiples of a random distribution.

**Figure 11:**
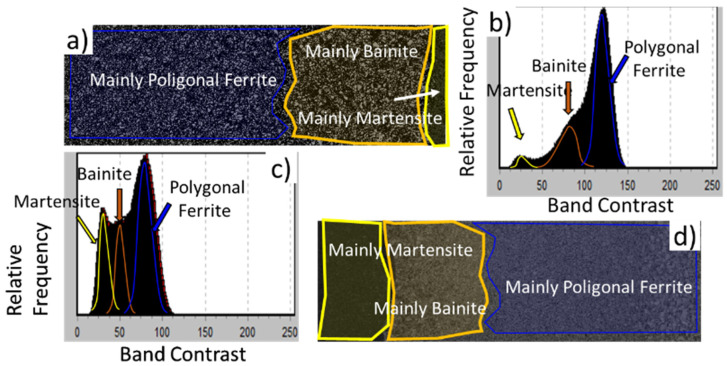
Detail of the microstructure in the FZ and HAZ regions analyzed in the EBSD-IPF maps. In (**a**) and (**b**), sample 9; in (**c**) and (**d**), sample 2.

The formed microstructure observed was mainly lath-type, such as martensite or bainite, in agreement with the literature [11,19,32]. Figure 11 shows a large FZ and HAZ area and the BC distribution’s respective phase discretization. The lower the heat input, the wider the martensitic FZ-HAZ microstructure and the smaller the CGHAZ.

**Figure 12:**
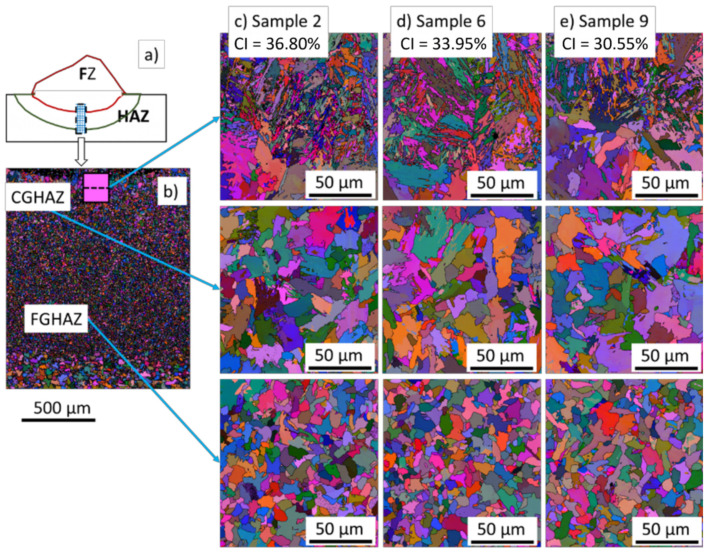
Euler maps with the transition between FZ, CGHAZ, and FGHAZ. In (**a**), schematic macro view of the Euler maps; in (**b**) a low macro magnification IPF showing the detailed zoomed arear from (**c**) to (**e**). FZ/CGHAZ interface, CGHAZ, FGHAZ of: (**c**) sample 2, (**d**) sample 6, and (**e**) sample 9.

Figure 12 shows the FZ and HAZ of the clad region for samples 2, 6, and 9. The CGHAZ consisted of coarse grains, mainly polygonal, with a few of them presenting the formation of a few martensite and lath structures inside, as observed in Figure 12c–e. The FGHAZ consisted of equiaxed grains ranging from 2 µm to 20 µm.

**Figure 13:**
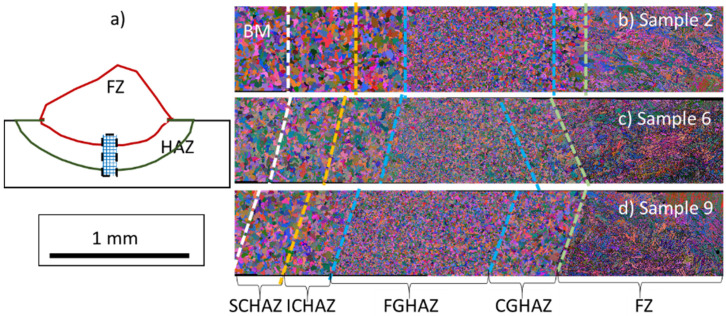
IPF maps showing all regions (from right to left): FZ, CGHAZ, FGHAZ, ICHAZ, SCHAZ, BM. In (**a**), schematic macro view of the IPF maps; in (**b**) sample 2, (**c**) sample 6, and (**d**) sample 9.

Figure 13 shows that the length of the produced HA zones was different for each condition. The length of the FGHAZ was the largest region of the HAZ for all cases. The CGHAZ and FGHAZ were the largest in sample 9 due to the higher heat input. The ICHA and SCHA zones were similar for all studied conditions. Furthermore, HAZ had an approximate length of 2 mm for samples 2, 6, and 9.

**Figure 14:**
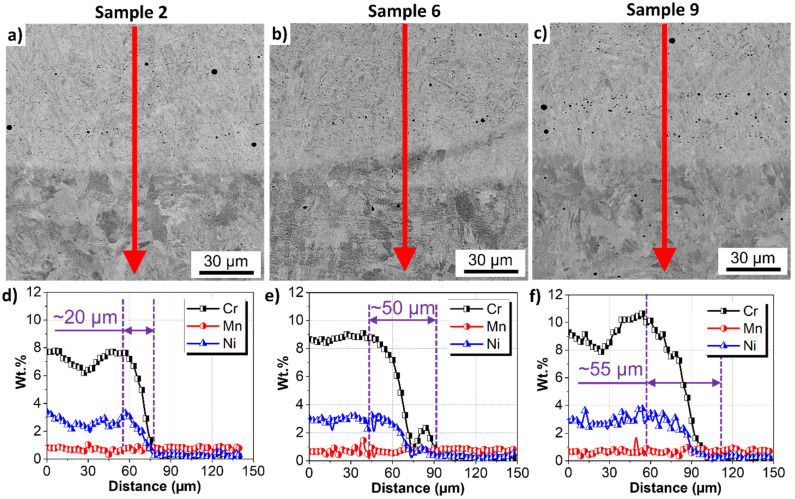
Chemical analysis of the clads. EDXS linescans for (**a**,**d**) sample 2; (**b**,**e**) sample 6, (**c**,**f**) sample 9. Cr, Mn, and Ni are shown, and Fe was identified as the remaining element.

The deposited clad (stainless steel) and the substrate (mild steel) had different chemical compositions (Table 1). Figure 14 shows the interface between FZ and HAZ. The cladding leads to a small substrate dilution, typical of fusion welding processes. The EDXS linescans for samples 2, 6, and 9 in Figure 14 show that the dilution zone was composed of a region ranging from 20 µm to 55 µm, where the gradient in Cr and Ni was present. The dilution was larger for sample 9, as indicated by the purple arrows in Figure 14. The variation of Cr content in the FZ for the investigated zone, more visible in Figure 14a–c indicates that the weld pool solidified rapidly. The Fe from the substrate did not have time to be diluted in the molten homogeneously deposited clad.

### 3.2. Statistical Analysis

The results of weld bead morphology relating the welding parameters with the resulting morphology and weld bead characteristics are presented in Table 5.

Statistical analyses were carried out for the parameters obtained in all clads produced according to the L9 orthogonal array shown in Table 5. The analysis was performed using ANOVA obtained through regression analysis for response variables. Regarding the effect of the variables, there was significant variation between the parameters for the same factor with a notable *p*-value, lower than the level of significance (0.05), rejecting the null hypothesis. Therefore, when the lines have a slight variation, there is also a *p*-value higher than the significance level, thus concluding that this particular input variable does not significantly influence the response. Table 6 shows that the average current was the most critical factor in controlling the related parameters, followed by welding speed and CTWD. Finally, no statistical relation was found between pulse frequency and the used parameters for a significance level of 0.05.

For the statistical generation of each response variable, the following equations for mathematical relationships were formulated.

Width (mm):

b=1.49+0.0317·x1+0.0335·x2 − 0.00792·x3+0.122·x4

Reinforcement (mm):

r=10.748 − 0.00894·x1 − 0.0484·x2 − 0.00735·x3 − 0.0511·x4

Penetration (mm):

p=2.670+0.00653 x1 − 0.0378 x2 − 0.00183 x3 − 0.0108 x4

Penetration area (mm²):

Ap=2.340+0.0469·x1 − 0.107·x2 − 0.0102·x3+0.142·x4

Reinforcement area (mm²):

Ar=59.200+0.0240·x1 − 0.215·x2 − 0.0879·x3 − 0.0780·x4

Dilution (%):

D=−1.164+0.00760·x1 − 0.00818·x2+0.00523·x3+0.0308·x4

Convexity Index (%):

CI=146.400−0.229·x1 − 0.453·x2−0.00545·x3−1.0200·x4

### 3.3. Hardness Measurements

**Figure 15:**
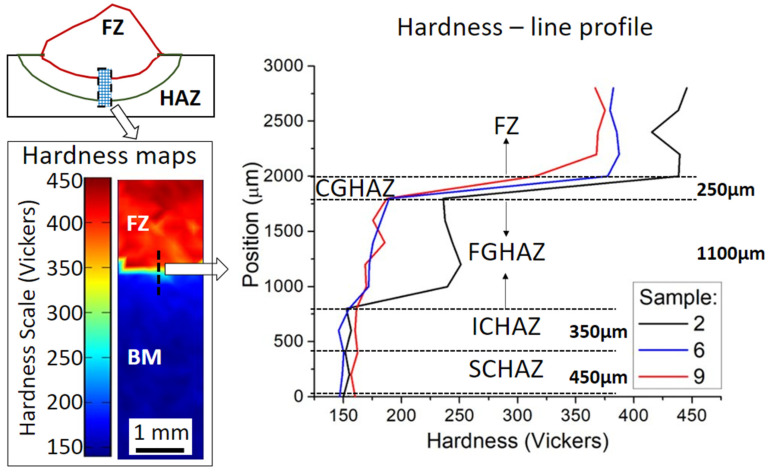
Hardness distribution in a 3 mm range of transition between the fusion zone (FZ) and base metal (BM).

Figure 15 shows at the left side an illustrative mapping of microhardness in sample 2 and the correspondent hardness line profiles extracted along the region covered by the dashed black line for each selected sample. The covered regions were FZ, HAZ, and a small part of BM, with a 3 mm measurement length. The three measured samples showed similar hardness profiles, although sample 2 showed the most elevated hardness values in the FZ and FGHAZ. The present phase fractions and the average cooling rates were directly related to the hardness values. For a high applied current and greater heat input, high hardness values were obtained in FZ, CGHAZ, and FGHAZ due to the presence of martensite and bainite. In sample 2, with the lowest heat input and therefore subjected to the fastest cooling rates, the martensitic fraction was clearly larger, as displayed in Figure 11, it causes the hardness peak (440 HV) in the FZ. Owing to the rapid cooling, the FGHAZ became more fine-grained in sample 2, as indicated in Figure 11c by a partition of polygonal ferrite with lower average band contrast (pattern quality) than in sample 9 in Figure 11b. This resulted in increased hardness of the FGHAZ (240 HV) when the lowest heat input was in sample 2.

The obtained hardness line profiles for the pulsed FCAW strategies agreed well with the literature for fusion welding techniques. Cladding with 410NiMo deposited by thermal spray in CA6NM martensitic stainless steel achieved microhardness values between 311 and 431 Vickers [36]. The hardness of a similar alloy, UNS S41500, electron beam welded was between 300 to 400 Vickers [37]. Hardness values between 260 and 350 Vickers in the fusion zone of an ER 41NiMo deposited onto an X20Cr13 martensitic stainless steel after multiple repair welding [38]. In addition, an autogenous single pass electron beam welded CA6NM exhibited hardness values between 320 to 400 Vickers in the weld seam [39]. Furthermore, weld seams of 13Cr-4Ni weld seam showed hardness values between 300 and 500 Vickers [24].

### 3.4. Residual Stresses

Residual stresses may be determined across weldments by several methods, such as diffraction techniques, hole drilling, and global contour methods [40,41]. Hole drilling is quick, simple, portable, and adequate for a wide range of materials, including non-crystalline and electric insulators, and allows for investigating thick section workpieces by deep hole drilling. However, the disadvantages include the interpretation of data, its destructive character, and the limited strain sensitivity and resolution.

Contour methods enable the determination of high-resolution maps of stress normal to the surface in a wide range of materials. The main requirement is that the planar section must be cut carefully through the material under residual stresses. Mechanical methods tend to create cutting stresses and reduce out-of-plane deflections while being measured. Hence, electric discharge wire machining (EDWM) is commonly used to diminish artifacts. The residual stresses can be determined by imposing the measured displacements with the negative sign within a Finite Element model. However, EDWM cannot be utilized to cut non-conductive materials. The limitations of the contour method involve its destructive nature and the interpretation of data. It considers that the residual stress state is independent of the length coordinate (i.e., a plane eigenstrain state exists). EDWM sectioning also ignores displacements of material points parallel to the cutting plane. It would require subsequent sectioning through different planes to reconstruct multiple stress components.

**Figure 16:**
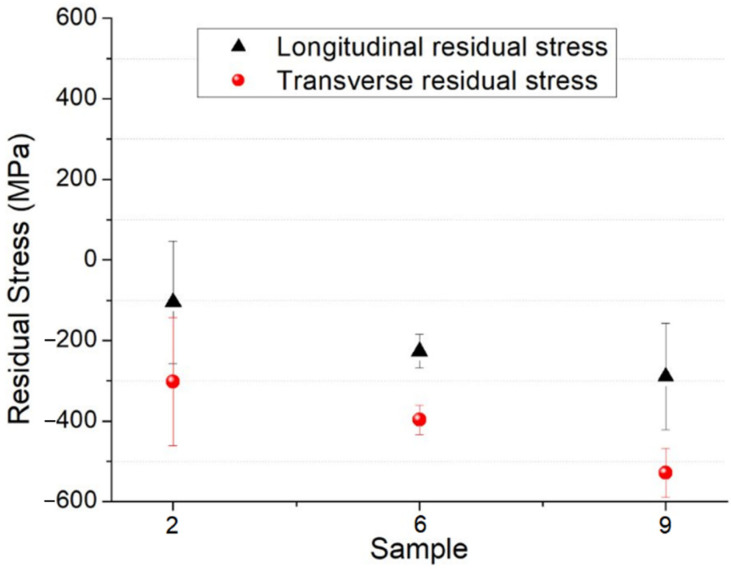
Residual stress values for all the three studied samples.

Lab X-ray stress analyses are versatile, phase selective, suit a wide range of crystalline materials, assess the multiaxiality of the stress state, and enable the separation of macro-and micro-residual stresses. However, X-ray diffraction yields information from only the near-surface region. In the present case of claddings, this is more relevant because clads of martensitic stainless steels are used for wear and corrosion protection. Hence, compressive stresses in the surface region with a few micrometers control wear, corrosion, and even fatigue resistances in these materials. Figure 16 displays the residual stress values on top of the clad in the longitudinal (parallel to the weld bead) and transverse (perpendicular to the weld bead) directions (Figure 5), as determined by X-ray diffraction.

The nature of the mean residual stresses in the clads was compressive. Results showed higher compressive stress (negative values) in the transverse direction than in the longitudinal direction in all cases. The residual compressive stress values in the transverse direction increased from −302 MPa for sample 2 to −397 MPa for sample 6, and finally −529 MPa for sample 9, thus exhibiting an inverse proportionality to the heat input. Therefore, the higher the heat input, the more compressive the residual stresses.

## 4. Discussion

One of the challenges of welding for cladding is the proper adjustment of parameters used in the process and their effects on the properties of the weld. For the investigated clads, the deposition rate decreased to a lesser extent with the increase of the average current and more significantly with the increased welding speed. Contrastingly, the weld bead images suggested that the deposition rate increased by increasing the pulse frequency and CTWD.

The ANOVA results demonstrate that the average current was the most critical factor in controlling the bead geometry. Higher current means a more pronounced Joule effect causing heating at the tip of the electrode. The higher the current, the larger is the weld width. The welding speed and CTWD also influenced the bead geometry. A lower deposition rate promotes a lower CI, while lower CI better suits the reinforcements and coating properties. The parameter study shows that fast welding speeds and low currents reduce the deposition rate. However, low currents result in high CI due to the lack of fusion and rapid solidification, tending to a circular cross-section morphology, as obtained in sample 2. However, cladding and welding applications should achieve the highest possible deposition rate, i.e., productivity.

The final welding parameters influenced the thermal history and, thus, the phase transformation kinetics in the produced clads. The welding pool transforms to austenite and δ-ferrite upon solidification. The δ-ferrite decomposes into austenite at temperatures not far from the solidification one. Austenite, then, undergoes a solid-state change to a structure that depends on both the cooling rate and the hardenability of the alloy, which is controlled by its chemical composition. No δ-ferrite was observed along the grain boundaries, in agreement with the literature [42,43], since in low-alloyed steels, δ-ferrite rapidly transforms into austenite during cooling.

**Figure 17:**
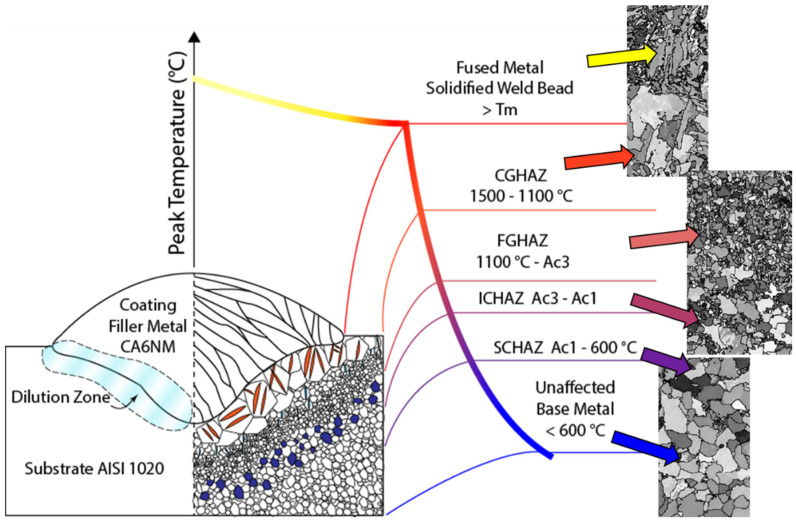
Schematic representation of welded microstructures and their metallurgical zones, divided according to the peak temperature. Images of the generated microstructure are displayed accordingly.

The heat-affected zone (HAZ) formed adjacent to the fusion zone. As indicated in Figure 13 and Figure 17**,** the HAZ can be subdivided into four regions according to the extent of grain growth and austenitization: coarse-grained zone (CGHAZ), fine-grained zone (FGHAZ), intercritical zone (ICHAZ), and over-tempered base metal [44]. They all presented large martensite and bainite phases formed in regions where the highest and intermediate cooling rates were achieved during the process. In addition, the FZ exhibited strong preferential crystallographic orientation, i.e., texture, which is a characteristic of columnar grains after fusion processes.

The present phases formed after welding governed the hardness values, which steeply increased due to the FCAW process. The higher the applied current, the lower is the hardness in FZ, CGHAZ, and FGHAZ, Figure 15. Besides, a larger weld bead width was obtained in sample 9, meaning a wider area of contact and heat transfer to the substrate, promoting a more intense mixture with the substrate material, i.e., dilution. On the other hand, sample 2 had the lowest current, leading to lower penetration and width, thus, low dilution. The higher dilution for sample 9 compared to sample 2 was visible in the EDXS linescans in Figure 14. The dilution of the substrate led to the incorporation of Fe into the clad (FZ), as indicated by the lower Cr content in regions of the FZ near the interface, Figure 14. It decreases the solid solution strengthening effect of the Cr and Ni. Thus, the higher the dilution, the lower the decrease in hardness of the filler metal CA6NM.

Consequently, sample 2 showed more elevated hardness values in FZ than samples 6 and 9. The welding speed also influenced the heat input, final bead morphology, and, consequently, hardness. A slow weld speed can provide a wider bead, higher heat input, and higher dilution. Figure 11 shows that the CGHAZ of sample 9 was the largest among the investigated samples due to the highest heat input compared to the other conditions. Finally, a transition of hardness values was observed due to dilution and mixture of chemical compositions between filler metal and substrate.

The dilution also affects the morphology of the deposition, promoting a broader area of contact. The ideal convexity index should be around 30% [45] to a standard acceptable deposit, and the conditions of interest range from 170, 200, and 230 A in pulsed current, with frequencies between 18 and 22 Hz. Unlike welding penetration, the lowest possible penetration with reinforcement and width compatible with the application is desired in cladding by welding. Thus, the larger the bead width, the fewer passes are necessary to coat a given area.

Steels with martensitic steel clads produced by the GTAW and FCAW welding processes generally present a segregated heterogeneous molten zone with well-defined austenitic and martensitic regions [34]. The welding parameters govern the phase transformation sequences and/or kinetics, phase distribution, and morphology. Moreover, complex thermal history and gradients during welding promote residual stresses [35,44]. Due to the different expansion and contraction rates of weld metal and HAZ, residual stresses can modify the crack propagation rates. Sample 2 showed the lowest residual compressive stress and the highest formation of martensite and largest CGHAZ, obtaining similar results [42], although there were contrary reports for the same alloy [46]. All samples presented compressive residual stresses at the clad surface, which is favorable to mechanical properties [9,47]. The highest heat input in sample 9 led to broader peak temperatures and temperature gradients, thus, higher induced thermal stresses during the fast cooling. The thermal stresses were caused by distortion in the crystal lattice that generated compressive stresses at the clad surface. The highest compressive residual stresses in sample 9 also agreed with the coarsest grains in the CGHAZ, indicating that this zone was subjected to higher peak temperatures.

The formed microstructure was mainly lath-type, such as martensite and bainite, in agreement with the literature [44,46,48]. A distinction of phases using BC from EBSD data has also been reported to enable the differentiation among martensite, bainite, and polygonal ferrite, as a result of increasing BC, respectively [49]. Figure 10 and Figure 11 show the EBSD maps and the band contrast with large FZ and HAZ areas, consistent with the BC phase discretization. The larger the FZ, the more significant the martensite fraction. Sample 2 shows the highest hardness values in the FGHAZ, Figure 15. The BC phase discretization indicates that the polygonal ferritic partition in sample 2 had a worse pattern quality due to a more significant amount of lattice imperfections when compared to sample 9. Thus, the higher hardness values for sample 2 in the FGHAZ are explained by grain refinement of the ferritic fraction formed in that region. The higher the heat input, the lower the hardness in the CGHAZ, Figure 15, due to the decrement of the martensite fraction as a result of slower cooling. Since in the ICHAZ and SCHAZ polygonal ferrite is the predominant phase and both have similar BC, as shown in Figure 11, the hardness values for samples 2, 6, and 9 in Figure 15 were similar in those two regions for the three samples.

## 5. Conclusions

The present work investigated the influence of the pulsed FCAW welding parameters on the final clad morphology, residual stresses, microhardness distribution, and microstructural gradients through the base metal, heat-affected zones, and fusion zone consisting of the martensitic AWS 410NiMo stainless filler metal. The results allow for drawing the following conclusions:

- Extensive martensite and bainite formation with preferential crystallographic orientation along the direction of heat extraction in the FZ, which is characteristic of columnar grains after fusion welding, were verified in all tested conditions.
- The different hardness distributions presented an inversely proportional relationship to the applied heat input during cladding. Heat inputs of 0.43, 0.47, and 0.79 kJ·mm^−1^ resulted in, respectively, 440, 387, and 368 Vickers hardness in the fusion zone near the interface. This occurred because lower heat inputs led to lower dilution, lower cooling rate, and, consequently, lower hardness values in the region near the interface in the FZ.
- The higher the heat input, the larger the grains and the more pronounced is the CGHAZ.
- A higher heat input led to higher compressive residual stress due to higher peak temperatures and broader temperature gradients. Those effects lead to higher thermal stresses, which cause lattice deformation, thus visible as compression stresses at the clad surface.
- The formation of bainitic microstructure in the CGHAZ zone is suggested by a decrement in the band contrast and was more visible in sample 9. Furthermore, it decreased the overall hardness values in the CGHAZ for samples 6 and 9.
- Lower heat inputs lead to lower peak temperatures, rapid cooling rates, and a more refined microstructure in the FGHAZ. In addition, the fine-grained ferrite with low quality of Kikuchi patterns leads to higher hardness values in the FGHAZ, the lower the heat input.
- Higher heat input is more advantageous to clad AWS 410NiMo stainless steel onto low carbon AISI 1020 steel by FCAW due to the low convexity index and smooth hardness transition between FZ and HAZs in addition to the more expressive compressive stresses.

## Figures and Tables

**Table 1:** Chemical composition of the materials used in wt%.

Material	C	Mn	Si	P	S	Cu	Cr	Ni	Sn	Mo
SAE 1020	0.21	0.49	0.19	0.03	0.035	0.20	0.15	0.14	0.06	---
410NiMo	0.027	0.59	0.44	0.024	0.006	---	12.5	4.86	---	0.43

**Table 2:** L9 Orthogonal array of the input variables.

Sample	Im (A)	Pulse Frequency (Hz)	v (mm·s^−1^)	CTWD (mm)	Irms (A)	Urms (V)	Q J·mm^−1^
1	170	18	5.0	30	193.3	15.2	470
2	170	22	5.8	33	185.5	17.0	430
3	170	20	6.7	36	160.6	17.8	340
4	200	18	5.8	36	191.4	25.5	670
5	200	20	5.0	33	182.2	26.4	770
6	200	22	6.7	30	178.3	22.2	470
7	230	18	6.7	33	209.9	27.8	700
8	230	20	5.8	30	215.4	25.4	750
9	230	22	5.0	36	195.3	25.3	790

**Table 3:** Experimental layout (L9) orthogonal array and measured welding geometry.

Sample	Weld Bead Height (mm)			Weld Bead Width (mm)			Penetration Depth (mm)			Convexity Index (%)
	r1	r2	rAV	b1	b2	bAV	p1	p2	pAV	
1	4.75	4.35	4.55	9.24	8.14	8.69	1.97	2.05	2.01	52.36
2	3.40	3.25	3.33	8.98	9.12	9.05	1.98	1.59	1.79	36.80
3	4.11	4.20	4.16	8.13	7.92	8.02	1.88	1.86	1.87	51.87
4	3.71	3.65	3.68	10.48	9.86	10.17	2.82	2.50	2.66	36.18
5	4.11	4.09	4.10	10.69	11.04	10.87	2.34	2.69	2.52	37.72
6	3.32	3.27	3.30	9.81	9.63	9.72	2.07	2.48	2.28	33.95
7	3.52	3.14	3.33	9.75	10.04	9.90	2.08	2.13	2.11	33.64
8	3.56	3.61	3.59	10.06	10.13	10.10	2.54	2.55	2.55	35.54
9	3.42	3.59	3.51	11.46	11.51	11.49	2.30	2.15	2.19	30.55

**Table 4:** Schematic morphology of the weld beads to the parameters selected. Where: penetration depth (p), reinforcement height (r), bead width (b), and convexity index (CI).

Sample	Welding Parameters	Schematic View of the Cross-Section
2	I_m_: 170 A; PF: 22 Hz; *v*: 5.8 mm·s^−1^; CTWD: 33 mm; CI: 36.80%; Q: 430 J·mm^−1^	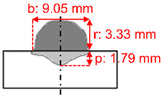
6	I_m_: 200 A; PF: 22 Hz; *v*: 6.7 mm·s^−1^; CTWD: 30 mm; CI: 33.95%; Q: 470 J·mm^−1^	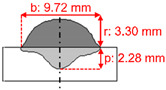
9	I_m_: 230 A; PF: 22 Hz; *v*: 5.0 mm·s^−1^; CTWD: 36 mm; CI: 30.55%; Q: 790 J·mm^−1^.	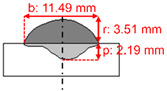

**Table 5:** Results of weld bead morphology with pulsed current. Where: I_m_, average current (A); PF, pulse frequency (Hz); v, welding speed (mm·s^−1^); CTWD, contact-tip-workpiece distance (mm); r, reinforcement height (mm); b, bead width (mm); p, penetration depth (mm); Ar, reinforcement area (mm^2^); Ap, penetration area (mm^2^); D, dilution (%); CI, convexity index (%).

Sample	Welding Parameters				Results						
	I_m_	PF	v	CTWD	b	r	p	Ap	Ar	D	CI
2	170	22.22	5.83	33	9.05	3.33	1.79	8.77	27.96	2.72	36.79
6	200	22.22	6.67	30	9.72	3.30	2.28	11.37	30.95	2.36	33.95
9	230	22.22	5.00	36	11.49	3.51	2.19	8.50	23.99	3.08	30.54

**Table 6:** Analysis of Variance (ANOVA) obtained through regression analysis for response variables (*p*-value).

Factor	b	r	p	Ap	Ar	D	CI
x1: Average Current (A)	0.000	0.000	0.040	0.009	0.103	0.023	0.000
x2: Pulse Frequency (Hz)	0.706	0.094	0.391	0.647	0.308	0.854	0.470
x3: Welding Speed (mm·min−1)	0.043	0.000	0.306	0.288	0.000	0.011	0.045
x4: CTWD (mm)	0.058	0.014	0.712	0.371	0.578	0.315	0.027

## Data Availability

The data that support the findings of this study are available from the corresponding author upon reasonable request.
